# Resolution in super-resolution microscopy – facts, artifacts, technological advancements and biological applications

**DOI:** 10.1242/jcs.263567

**Published:** 2025-05-27

**Authors:** Kirti Prakash, David Baddeley, Christian Eggeling, Reto Fiolka, Rainer Heintzmann, Suliana Manley, Aleksandra Radenovic, Hari Shroff, Carlas Smith, Lothar Schermelleh

**Affiliations:** ^1^ Delft Center for Systems and Control, Faculty of Mechanical, Maritime, and Materials Engineering, Technische Universiteit Delft, Delft, 2628 CN, The Netherlands; ^2^ Auckland Bioengineering Institute, University of Auckland, Auckland, 1010, New Zealand; ^3^ Institute of Applied Optics and Biophysics and Abbe Center of Photonics, Friedrich-Schiller-University Jena, Jena, 07745, Germany; ^4^ Leibniz Institute of Photonic Technology, Jena, 07743, Germany; ^5^ Lyda Hill Department of Bioinformatics, University of Texas Southwestern Medical Center, Dallas, TX 75390, USA; ^6^ Institute of Physical Chemistry and Abbe Center of Photonics, Friedrich-Schiller-University Jena, Jena, 07745, Germany; ^7^ Laboratory of Experimental Biophysics, School of Basic Sciences, Institute of Physics Interfaculty Institute of Bioengineering, EPFL SB-LEB, Lausanne, 1015, Switzerland; ^8^ Laboratory of Nanoscale Biology, School of Engineering, Institute of Bioengineering, EPFL STI IBI-STI LBEN, Lausanne, 1015, Switzerland; ^9^ Janelia Research Campus, Howard Hughes Medical Institute (HHMI), Ashburn, VA 20147, USA; ^10^ Department of Biochemistry, University of Oxford, Oxford OX1 3QU, UK

**Keywords:** Super-resolution microscopy, Resolution, Contrast, Stimulated emission depletion, Structured illumination microscopy, Single-molecule localization microscopy

## Abstract

Super-resolution microscopy (SRM) has undeniable potential for scientific discovery, yet still presents many challenges that hinder its widespread adoption, including technical trade-offs between resolution, speed and photodamage, as well as limitations in imaging live samples and larger, more complex biological structures. Furthermore, SRM often requires specialized expertise and complex instrumentation, which can deter biologists from fully embracing the technology. In this Perspective, a follow-up to our recent Q&A article, we aim to demystify these challenges by addressing common questions and misconceptions surrounding SRM. Experts offer practical insights into how biologists can maximize the benefits of SRM while navigating issues such as photobleaching, image artifacts and the limitations of existing techniques. We also highlight recent developments in SRM that continue to push the boundaries of resolution. Our goal is to equip researchers with the crucial knowledge they need to harness the full potential of SRM.

## Teaser

**Summary:** Super-resolution microscopy (SRM) has emerged as a powerful tool for biological discovery. This paper compiles expert opinions on crucial, yet often overlooked, aspects of SRM that are essential for maximizing its benefits and advancing the field.

## Introduction

Fluorescence light microscopy has long been a foundational tool in the life sciences, contributing to countless key discoveries by allowing scientists to directly visualize and measure cellular structures and processes. However, it has been historically constrained by the diffraction limit, first described by Ernst Abbe in 1873, which restricts optical resolution to about half the wavelength of the light used (Abbe, 1873; Rayleigh, 1880). In modern fluorescence microscopy, this translates to a resolution limit of ∼200–300 nm in the lateral directions (*x* and *y*), and 500–800 nm along the optical axis (*z*). In the past two decades, advances in super-resolution microscopy (SRM) have overcome this limitation, pushing spatial resolution into the 10–150 nm range. These developments have significantly narrowed the resolution gap between fluorescence microscopy and electron microscopy (EM), opening new possibilities for biological research (Liu et al., 2022; Schermelleh et al., 2019).

The current commercially available far-field epifluorescence SRM techniques can be broadly categorized into four types (Table 1). First, pixel reassignment image scanning microscopy (ISM) methods (such as Re-scan, AiryScan, SoRA and iSIM, see Glossary) are extensions of single- or multi-point laser scanning confocal microscopy (see Glossary) that offer a moderate ∼1.4-fold improvement in *xy*-resolution beyond the classical diffraction limit (Sheppard, 2021), which can be further extended in *xy* and *z* after deconvolution (see Glossary). Second, linear structured illumination microscopy (SIM) uses patterned illumination to generate a moiré interference that encodes extended-resolution information from the sample. This information can be computationally extracted from the raw data to reconstruct an image with up to two-fold resolution improvement in lateral and axial directions (Gustafsson et al., 2008; Chen et al., 2023). Third, stimulated emission depletion (STED) microscopy uses a doughnut-shaped depletion beam superimposed upon a confocal scanning laser beam to manipulate the point spread function (PSF, see Glossary) and force molecules to emit fluorescence from a smaller volume, allowing for greater spatial resolution (Hell, 2007). The resolution increase offered by STED depends on the intensity and shape of the depletion beam and is typically limited by the signal-to-noise ratio (SNR) rather than diffraction.

**Glossary**

**Axial resolution:** the ability of a microscope to differentiate between closely spaced objects along the optical axis.

**Peak signal-to-noise ratio (PSNR):** a metric used to measure the quality of an image by comparing the maximum possible signal to the level of background noise.

**Spectral signal-to-noise ratio (SSNR):** a frequency-domain metric that quantifies the signal-to-noise ratio as a function of spatial frequency.

**Confocal microscopy:** a laser scanning microscopy technique that improves optical resolution and contrast and enables optical sectioning for 3D imaging by suppressing the detection of out-of-focus light using spatial filtering.

**Contrast or modulation transfer function (CTF or MTF):** the CTF, or related MTF, describe the ability of an optical system to reproduce contrast at different spatial frequencies. The MTF is a subset of the optical transfer function [OTF, the Fourier transform of the point spread function (PSF)] that considers only its magnitude.

**Deconvolution:** a computational method that uses knowledge about image formation and often prior knowledge of the sample to restore images by partially reversing the blurring process, improving resolution and image quality.

**Diffraction limit (Abbe limit):** the inherent limitation in resolution for conventional microscopy due to the wave nature of light, defined by a formula for the theoretical limit of resolution in optical systems, stating that resolution is limited to half the wavelength of light used for imaging at the sample.

**Direct stochastic optical reconstruction microscopy (dSTORM):** a type of SMLM where reversible switching of fluorophores between fluorescent and dark states (‘blinking’) is used to achieve super-resolution imaging.

**Expansion microscopy (ExM):** a technique that physically expands fixed biological samples using polymer embedding and controlled swelling to increase spatial resolution.

**Fourier ring correlation (FRC):** a quantitative method used to measure the resolution of super-resolution images. FRC assesses the spatial frequency content of an image, determining the highest frequencies that can be accurately resolved and providing a quantitative measure of resolution improvement compared to conventional microscopy techniques.

**Fourier domain (also called Fourier space, Frequency space or Frequency domain):** the coordinate space in which the spatial frequency content of an image is represented after applying a Fourier transform.

**Image contrast:** the difference in intensity or color between an object and its background in an image. This potentially has multiple overlapping definitions. A strictly technical definition refers to a periodic structure with zero background.

**Image scanning microscopy (ISM):** also referred to as point-scanning SIM, is a collective term for confocal microscopy techniques that acquire data using multiple adjacent (small) pinholes in parallel, usually via a detector array, and algorithmic reassignment of fluorescence signal to increase the lateral resolution by a factor of ∼1.4, which can be further improved by deconvolution (e.g. Airyscan, iSIM).

**Instant structured illumination microscopy (iSIM):** an ISM technique that uses multi-point scanning with micro-optics arrays and camera detection to increase sensitivity and speed.

**Lateral resolution:** the ability of a microscope to distinguish between closely spaced objects in the plane perpendicular to the optical axis.

**Localization precision:** the precision in determining the position of a fluorescent molecule in SRM, often used in SMLM as a measure of resolution. Owing to the sampling criterion, the resolution cannot be better than ≥2 times the localization precision. In practice, this is further limited by the specificity of photoswitching or blinking events and labeling density (Lelek et al., 2021).

**MINFLUX:** an emerging SRM technique that establishes the coordinates of a molecule with (minimal) emission fluxes, originating from a local excitation minimum to achieve a resolution at the nanometer scale. Related modulation-enhanced SMLM methods are SIMFLUX, SIMPLE, ModLoc, ROSE, COLD and RESI microscopy.

**Non-linear SIM:** a form of SIM that provides higher resolution by using a fluorescent response that depends non-linearly on the intensity of the illuminating light. This can, for example, be achieved by saturating the excitation process or inducing photoswitching.

**Numerical aperture (NA):** the product of the refractive index of the medium embedding the sample and the sine of the half opening angle of the cone of light transmitting the detection system. The NA helps to quantify the light-gathering capacity and resolution of an objective lens in a microscope.

**Photobleaching:** a process wherein fluorophores lose their ability to fluoresce due to exposure to light, impacting image quality over time.

**Point spread function (PSF):** the image of a single point-like object generated after the process of imaging. In approximation, an imaging system performs a convolution of the object density with the PSF. The frequency-domain representation of the PSF is called optical transfer function (OTF), which represents the ability of an optical system to transfer spatial frequencies from the object to the image.

**Sampling criterion (Nyquist criterion):** a principle stating that to interpret a signal unambiguously the sampling rate must be at ≥2 times the maximal frequency present in the signal.

**Single-molecule localization microscopy (SMLM):** umbrella term for super-resolution techniques that localize individual fluorescent molecules to reconstruct high-resolution images, such as PALM, STORM, dSTORM or DNA-PAINT.

**Spatial frequency:** the number of changes in a pattern that occurs per unit distance, often used in analyzing image content in microscopy.

**Spatial resolution:** the level of detail or smallest discernible feature in an image obtained through the microscope.

**Structural resolution:** the ability to resolve structural features within a biological sample, typically equivalent to the range of frequencies that is above the noise floor in Fourier space.

**Super-resolution optical fluctuation imaging (SOFI):** a technique that enhances resolution by analyzing temporal fluctuations in the emission of fluorophores. By analyzing multiple frames of a time-lapse sequence, SOFI reconstructs an image with improved resolution compared to conventional methods.

**Total internal reflection fluorescence (TIRF):** an optical imaging technique that allows selective excitation of fluorophores near the glass-water interface, typically within 100–200 nm of the coverslip (evanescent field), by illuminating the sample at a supercritical (reflective) angle. Inclined excitation at a subcritical non-reflective angle is called highly inclined and laminated optical sheet (HILO) illumination and is often used to reduce background compared to straight (epi-)illumination.

**Widefield microscopy:** conventional microscopy where the entire specimen is illuminated evenly at once and the emitted light is detected by a camera, providing high sensitivity and temporal resolution at the expense of optical sectioning and (axial) resolution.

**Table 1.** Overview of the most common commercially available optical SRM imaging techniques

Technique (variants/synonyms)	Pixel reassignment ISM (Re-scan, AiryScan, iSIM, etc.)	SIM (linear SIM, SR-SIM)	STED	SMLM (dSTORM, PALM, PAINT, MINFLUX, etc.)
**General features**				
Super-resolution principle	Reduced Airy unit size detection and mathematical or optical reassignment of fluorescence for enhanced resolution reconstruction	Moiré interference by patterned illumination and mathematical reconstruction of frequency-shifted sample details	Effective point spread function reduction by superposition of standard excitation with a ‘donut-shaped’ depletion laser beam, exploiting dye photochemistry	Temporal separation of stochastic fluorescence emissions; fitting, filtering and summing individual emitter positions to create a binary image (‘pointillism’), exploiting dye photochemistry (dSTORM, PALM) or binding kinetics (PAINT)
Fluorescence illumination	Single- or multi-point laser scanning	Laser; widefield	Single-point laser scanning	Laser; widefield (MINFLUX: point scanning)
Fluorophore detection	Photon detector (array) or camera-based; ensemble	Camera-based; ensemble	Photon detector (array); ensemble	Camera-based; single-molecule (MINFLUX: photon detector)
Raw data	*xy*(*z*)-scans	9 or 15 raw images per plane	*xy*(*z*)-scans	>10,000 raw images, single plane (MINFLUX: ≥4 detections per molecule)
Image reconstruction	Yes	Yes	No	Yes
**Spatial resolution**				
Diffraction-limited	Yes	Yes	No	No
Wavelength-dependent	Yes	Yes	No	No
*xy* localization precision	N/A	N/A	N/A	10–20 nm (MINFLUX: 1–5 nm)
*xy* resolution*	140–180 nm	90–130 nm	∼50 nm (2D STED) ∼100 nm (3D STED)	≥ 2× lower than either localization precision or labeling density^‡^
*z* resolution	Diffraction-limited	Diffraction-limited (2D-SIM) 250–400 nm (3D-SIM)	Diffraction-limited (2D STED) ∼100 nm (3D STED), tuneable in exchange for improved *xy* resolution	Additional localization in Z is possible; however, with lower precision than and usually at the expense of the lateral localization precision
Added deconvolution	Routinely applied to further enhance resolution to 120-150 nm in *xy* and 350-500 nm in Z	Optional, to further enhance resolution to 60 nm in *xy* and 200 nm in *z*^§^	Routinely applied to compensate for low contrast (signal-to-background ratio)	No
High-frequency contrast transfer efficiency^¶^	Intermediate	High	Low	N/A
Resolution in theory limited by	NA and wavelength (λ) (∼1.4× diffraction limit, optically)	NA and λ (2× diffraction limit)	Depletion laser intensity and geometry	Photon count, density and separation of emitters
Effective resolution limited by …	Contrast	(Modulation) contrast, spherical aberration	Contrast, dynamic range, dye photostability	Buffer conditions (dSTORM), on–off binding (PAINT), detection bias
**Versatility**				
Optical sectioning or *z*-stack acquisition	Yes	Yes	Yes	Single-plane acquisition with 2D or 3D localizations
TIRF option for near-field applications	No	Yes	No	Yes
Temporal resolution (suitability for live cell imaging and throughput)	Low (single-point scanning) High (multi-point scanning)	High (2D-SIM, TIRF-SIM) Intermediate (3D-SIM)	Variable, dependent on field of view (low for cell-sized fields of view)	Very low (fixed cells only)
Photodamage (photobleaching and phototoxicity)	Intermediate (single-point scanning) Low (multi-point scanning)	Low (2D-SIM, TIRF-SIM) Intermediate (3D-SIM)	High (tuneable with decreased spatial resolution)	Very high (dSTORM) High (PALM, PAINT)
Multi-color (routine use)	4+ colors	3–4 colors	2–3 colors	2 (PALM) to multiple (PAINT)
Dye restrictions	Low	Low	Intermediate	High
Aberration sensitivity	Low	High	Intermediate	Intermediate
Imaging depth	High	Low	Intermediate	Low
Susceptibility to artifacts	Low	High	Low	High
Commercial implementations	Zeiss LSM 980 AiryScan 2, Nikon NSPARC, Yokogawa SoRa (multi-point scanning), VisiTech VT-iSIM (multi-point scanning), etc.	Zeiss Elyra 7 Lattice SIM, CSR Biotech MI-SIM, GE DeltaVision OMX SR (discontinued), Nikon N-SIM S (discontinued)	Leica STELLARIS TauSTED, Abberior MATRIX STED & STEDYCON	Nikon N-STORM, Zeiss Elyra 7 PALM, ONI Nanoimager, Bruker Vutara 352, Abbelight SAFe 360, Abberior MINFLUX
References	Wu and Shroff, 2018; Sheppard, 2021	Heintzmann and Huser 2017; Demmerle et al., 2017; Prakash et al. 2021	Vicidomini et al., 2018; Lukinavičius et al., 2024	Lelek et al., 2021; Power et al., 2024

For conciseness, the table does not include bespoke SRM techniques that are not (yet) commercially available, non-optical ExM approaches based on physical sample expansion, dual objective techniques (e.g. 4Pi, lattice light sheet) that increase resolution in only one dimension and fluctuation-based algorithmic approaches, such as SOFI and SRRF, which improve the temporal resolution of dSTORM-type blinking microscopy at the expense of spatial resolution. The references row highlights recent topical reviews or protocols paper. For comprehensive overview articles, see also references in the main text.

*Resolution values refer to routinely achievable resolutions with high-quality standard samples and on well-aligned commercially available systems.

^‡^In SMLM, spatial resolution depends on how precisely a dye molecule is localized and how densely such dyes are spaced. Even with high localization precision (e.g. 10 nm), sparse labeling (e.g. one dye molecule every 20 nm) limits resolution to ≥40 nm. Conversely, dense labeling alone is insufficient if localization precision is poor.

^§^Zeiss and CSR Biotech systems offer additional deconvolution options (‘SIM^2^’, ‘computational SR’). However, these carry increased risk of introducing deconvolution artifacts, such as over-sharpening or structural biasing.

^¶^Higher efficiency allows for rescuing of structural details even at reduced contrast (e.g. due to increased out-of-focus blur background contribution).

Of note, all ensemble fluorophore-detecting methods, ISM, SIM and STED can be further augmented by deconvolution algorithms, which overcome some of the limitations imposed by low SNR and further increase resolution, albeit at the expense of introducing non-linear representations of fluorescence intensities that might affect data quantification.

A fourth group, distinct from previous ensemble methods, comprise single-molecule localization microscopy (SMLM) techniques, such as photoactivated localization microscopy (PALM) (Betzig et al., 2006), stochastic optical reconstruction microscopy (STORM) (Rust et al., 2006; Hess et al., 2006) and points accumulation for imaging in nanoscale topography (PAINT) (Sharonov and Hochstrasser, 2006; Jungmann et al., 2010). These methods temporally isolate fluorophore ‘blinking’ or photoswitching events (in PALM or STORM, respectively), or transient binding events between target sites and labeling molecules, such as short oligonucleotide strands as in DNA-PAINT (Schnitzbauer et al., 2017) and generate a ‘pointillistic’ image by combining individual fluorescence emitter positions with a localization precision (see Glossary) that is dependent on the photon count. This requires the acquisition of extended raw image series, thus rendering SMLM inherently slow. SMLM techniques have proven particularly effective for localizing individual molecules down to ∼10 nm nanometer precision in fixed samples (Baddeley and Bewersdorf, 2018; Lelek et al., 2021). By capturing intensity variations at reduced light intensities followed by image correlation, as in super-resolution fluctuation imaging (SOFI, see Glossary; Dertinger et al., 2009) and super-resolution radial fluctuations (SRRF; Gustafsson et al., 2016), acquisition times can be significantly reduced to even allow live-cell imaging, albeit at the cost of significantly reduced resolution gain (Pawlowska et al., 2021).

More recent developments have focused on pushing localization precisions down to the Ångstrom range by combining SMLM with modulated illumination, such as in MINFLUX (see Glossary) and related methods (Balzarotti et al., 2017; Reymond et al., 2019; Gu et al., 2019; Cnossen et al., 2020; Mazal et al., 2022; Reinhardt et al., 2023). However, these improvements come with trade-offs, such as the demand for spatial and temporal sparsity – that is, fluorophores must be well-separated in space and activated sequentially over time to prevent signal overlap, which otherwise limits the ability to image extended structures or crowded environments (Kalisvaart et al., 2022; Gwosch et al., 2020; Prakash and Curd, 2023; Smith et al., 2024).

Of note, widefield (see Glossary)-based SIM and SMLM can be combined with total internal reflection fluorescence (TIRF, see Glossary), which is particularly useful for interrogating cell surface biology, or highly inclined laminar optical sheet (HILO) illumination (see Glossary), which reduces background in crowded sample environments. Single-particle tracking (SPT), which provides time-resolved tracks of molecular movements (and thus, strictly speaking, is not an image-generating method), has benefited from being combined with SRM principles to extend localization precision and temporal resolution (e.g. sptPALM and MINFLUX tracking) (Manley et al., 2008; Balzarotti et al., 2017). Finally, expansion microscopy (ExM, see Glossary), which involves physical expansion of biological samples by a factor of four or higher before imaging, enables access using conventional imaging to size scales that are usually hidden, even more so when combined with SRM (Wassie et al., 2019; Zhuang and Shi, 2023; Hümpfer et al., 2024). ExM combined with various forms of microscopy is gaining traction and has the potential to compete with the highest-resolving optical SRM techniques.

It is important to understand the trade-offs associated with SRM. Higher resolution typically requires longer acquisition times and increased illumination light dosage, which reduces temporal resolution and can lead to photobleaching (see Glossary) and phototoxicity in the sample. Techniques such as STED and SMLM are traditionally more prone to these challenges, as intense light exposure can damage live cells or limit the duration of observation due to photobleaching. Another limitation of many SRM techniques is their reliance on chemically fixed cells, which can raise questions about structural preservation and restrict their application for studying dynamic processes (Shroff et al., 2024; Prakash et al., 2024). Additionally, the sample volume that SRM methods can image is often restricted, typically to two-dimensional planes or small fields of view, making it difficult to apply these techniques to large, complex tissues or whole organisms (Schermelleh et al., 2019). Furthermore, techniques like SMLM are often limited to imaging sparse, isolated entities, such as vesicles or single molecules, rather than densely packed or highly dynamic systems.

With this increasingly complex imaging landscape in mind, we asked leading experts to contribute their opinions on some frequently asked questions in SRM (Box 1). Questions 1–5 are covered in our recent article (Prakash et al., 2024) while questions 6–11 are covered here (with the response from each expert denoted by initials). We hope these questions facilitate an open discussion regarding the limitations of SRM in the microscopy community and provide valuable insights for biologists already using or aiming to apply SRM for their research.

**Box 1. Frequently asked questions in super-resolution microscopy**

Covered in Prakash et al., 2024:

1. How would you define resolution?
2. What are the main trade-offs in microscopy?
3. What are the benefits and risks of computational post-processing?
4. What are the main effects of labeling on super-resolution microscopy?
5. Where do you see super-resolution microscopy developing into in the coming years?
  Covered here:
6. Which is more important to prioritize: axial or lateral resolution?
7. What is the relationship between image contrast and resolution?
8. What are the benefits and downsides of linear versus non-linear microscopy methods?
9. How does the potential of Ångstrom-resolution light microscopy compare to that of EM?
10. How can one accurately assess image quality and deal with artifacts in SRM?

## Which is more important to prioritize – axial or lateral resolution?

In microscopy, lateral (*d*_lateral_) and axial (*d*_axial_) resolution (see Glossary) are key to determining image clarity in 3D structures. However, for some experimental setups, one of these parameters might be more important than the other. The lateral resolution of a widefield fluorescence microscope is given by: *d*_lateral_=*λ*/(2*NA*), where *λ* is the wavelength of the emitted light and *NA* is the numerical aperture (see Glossary) of the objective lens. Axial resolution is expressed as: 
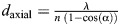
, where α is the half opening angle and *n* is the refractive index of the immersion medium. These equations highlight how both lateral and axial resolution depend on the wavelength of light and numerical aperture, with axial resolution typically being much lower and more challenging to improve. The resolution anisotropy (unevenness between the *xy* and *z* resolution) and the volumetric resolution are important factors to consider when imaging 3D objects.

**K.P.:** In the realm of 3D imaging, a rough rule suggests an approximate requirement that eight times more signal from the fluorophore is needed to attain a resolution comparable to that achieved in 2D imaging. In localization-based techniques, obtaining a sufficient signal density for 3D reconstructions is challenging, although DNA-PAINT might offer an advantage. Even with high signal density, improving the precision of localized molecules is essential. If precision is low, signals overlap, reducing the final resolution (Legant et al., 2016). However, there is also a trade-off between optimal lateral and axial resolution: for example, increasing lateral resolution by filtering out signals with the highest number of photons can lead to errors in the axial resolution estimates of 3D test samples (Prakash and Curd, 2023).

**D.B.:** This is hugely sample-dependent. Some samples need improved axial resolution, but there are many cases where two dimensions are sufficient – the wealth of data and discoveries using pre-tomographic EM are good evidence for this.

**C.E.:** This depends on the structure to be resolved. Imaging 3D structures, such as the cytoskeleton or organelles, requires good resolution in all directions. Axial resolution is less of an issue for imaging most membranes, yet investigations of membrane curvature or uptake mechanisms would also favor higher axial resolution. Therefore, it is advantageous to have all possibilities in hand, such as 3D-STED with varying STED laser power and confinement directions, MINFLUX with different laser patterns, SIM with different structural illumination schemes, or (d)STORM and PALM with their different 3D approaches.

**R.F.:** I would argue that the transition to biological investigations in 3D micro-environments requires a push towards improving isotropic spatial resolution – when the lateral and axial resolutions are equivalent – as there are no preferred spatial orientations of biological features of interest. Isotropic resolution is still a tough requirement to achieve, as the axial resolution is typically three to four times worse than the lateral. How this will be approached in the future – e.g. with interferometric opposing-lens (4Pi) setups, light-sheet microscopy, mirrored illumination, image restoration methods) – will be exciting to watch.

**R.H.:** This depends on the scientific question being addressed. The axial resolution is of little relevance for 2D flat membranes in the plane of focus. For 3D sample features, such as chromatin or mitochondria, the axial resolution, being typically the lowest, constitutes the weakest link and is therefore the most important limit to address. However, one also has to acknowledge that systems with a large gap between axial and lateral resolution essentially perform an extended *z*-projection within a single recorded image, which, depending on the problem at hand, can be useful by itself (Chang et al., 2023).

**S.M.:** Many biological studies have relied on 2D data to draw interesting conclusions. 3D data from powerful instruments, such as light sheet microscopes, is still sometimes mapped onto a 2D surface for easier handling and interpretation. Even so, I think that, as with any method, some questions can only be answered with true 3D data, which will require improvements in axial resolution. As SRM methods become more accessible, I am confident that there will be discoveries that follow from a shift in mindset for designing microscopy experiments to better acquire 3D data.

**C.S.:** This heavily relies on the scientific inquiry and the sample being studied. For example, when dealing with 3D structures and anisotropic micro-environments, a more significant emphasis should be placed on enhancing axial resolution. However, in situations where axial information holds less significance, such as imaging flat membranes, lateral resolution might take precedence. Therefore, researchers should take these considerations into account when crafting experimental designs and choosing suitable imaging techniques. Typically, one microscopy technique will stand out as the most suitable choice for a particular sub-inquiry. Researchers should also consider the trade-offs between imaging speed, resolution and phototoxicity. Microscopy techniques must strike a balance between these variables, underscoring the need for a nuanced approach. Although improved resolution, especially in the axial dimension, is desirable for comprehensive 3D imaging, it often comes at the cost of longer acquisition times and heightened phototoxicity. Researchers must evaluate the specific needs of their experiments, the characteristics of their samples and the potential impact on imaging speed and phototoxicity when making decisions about optimizing resolution in microscopy applications. This trade-off highlights the intricate interplay among various factors that influence the selection of microscopy parameters and emphasizes the significance of customizing imaging strategies to align with the requirements of each research endeavor.

**H.S.:** This is context dependent. As someone who does mostly 3D imaging, I think about axial resolution a lot. Often, I find that the axial resolution is the primary practical limit for my experiments rather than the lateral resolution limit.

**L.S.:** Biology, with few exceptions, is inherently 3D and dynamic. Although there are certainly cases where a 2D microscopic representation of a biological structure is sufficient, it is more typically the third and often the fourth dimension (i.e. time) that enables key advances in our knowledge. To identify and resolve biological targets within the context of extended 3D objects, the ability to achieve optical sectioning through efficient out-of-focus background removal is therefore equally as important as an increase in axial resolution. Both are essential for accurately capturing and interpreting the complexity of living systems in their native contexts.

**A.R.:** The choice between improving axial or lateral resolution depends on the specific research question. Among the many promising strategies for enhancing resolution in both dimensions are polarization-based SMLM techniques. These improvements can be achieved by engineering the point spread function (PSF) to increase localization precision (Ding and Lew, 2021), as well as by enhancing the signal-to-noise ratio (SNR) through polarization filtering and advanced optical sectioning methods (Rimoli et al., 2022).

## What is the relationship between image contrast and resolution?

In microscopy, contrast refers to the ability to distinguish objects or structural features within a sample based on their fluorescence intensity and discern them from the surrounding background, whereas resolution refers to the smallest distance at which objects can be separated. Translated to the Fourier domain, contrast is the visibility of structures with a given periodicity described by the contrast (or modulation) transfer function (CTF and MTF, see Glossary), whereas resolution is given by the maximum spatial frequency (see Glossary) the microscope can detect. In raw image data, contrast is best represented by the signal-to-background ratio (SBR). Unlike the SNR, which sometimes refers to detector noise alone, SBR also includes sample-specific contributions, such as autofluorescence, nonspecific labeling, light scattering and out-of-focus blur. Additionally, contrast can refer to the visual perception of intensity differences to the human eye, which can be enhanced via histogram stretching (by changing contrast and brightness display settings), false coloring and different rendering methods in image post-processing.

**K.P.:** In single-molecule localization microscopy (SMLM), image reconstruction has recently trended toward rendering with arbitrary localization precision for display rather than using the actual precision. This practice can create visually striking images with high contrast, but it risks misinterpreting resolution and potentially leading to erroneous conclusions about the data (Prakash and Curd, 2023). Additionally, color maps are often used to further emphasize bright signals against a dark background. However, when these visual enhancements are applied subjectively, they can potentially skew the perception of localization precision or labeling density (Prakash, 2022).

**C.E.:** Both are influenced by many factors, such as noise and labeling. They are also interrelated: for example, the best contrast will not create a great image without sufficient resolution and vice versa. Therefore, work on improving both!

**R.F. and H.S.:** Contrast can be seen as the visibility of structures with a given periodicity, which is described by the CTF or MTF of the microscope, whereas resolution is given by the maximum spatial frequency the microscope can detect, although there are nuances between the theoretical maximum spatial frequency and which frequencies rise above the noise floor. Traditionally, we microscopists push for the highest resolution. Nevertheless, there can be benefits to having a microscope system that provides good contrast for intermediate spatial frequencies, for example, in less-than-ideal imaging situations (e.g. high out-of-focus blur).

**S.M.:** Contrast and resolution are distinct properties of images that are both important for measuring features of interest. I would note that SRM methods that use non-linear optical transitions cannot be assumed to linearly map fluorophore density to signal intensity or contrast. This is important when imaging is used to dissect the relationship between protein abundance and structure or function, given that relative intensity might not be a proxy for abundance.

**C.S. and R.H.:** Image processing can adjust contrast to make structures stand out by changing brightness, contrast or applying filters. Resolution, however, depends on the design of the microscope and limits how much detail can be seen. Although contrast can be manipulated by postprocessing, improving resolution typically involves upgrading the microscope hardware or sample preparation. Resolution can be estimated from a single image using the single image versions of the Fourier ring correlation (FRC, see Glossary) and the spectral signal-to-noise ratio (SSNR, see Glossary) (Smith et al., 2021). The key here is that FRC provides a single value estimate for the maximum observable frequency, that is the spatial resolution, whereas the SSNR gives the SNR object as a function of spatial frequency. To ensure the accuracy of the super-resolution reconstruction, it is advisable to evaluate its consistency with the low-resolution reference. This can be quantitatively assessed using the resolution-scaled Pearson coefficient (Culley et al., 2018).

**L.S.:** Contrast and resolution are intricately linked. Without contrast, there is no ability to resolve structural features. It is important to recognize that contrast can vary from sample to sample, image to image, and even in different regions of the same image, as the level of background can vary locally. This is most notably caused by the amount of out-of-focus blur and light scattering in densely packed fluorescent features versus in isolated features surrounded by sufficient ‘empty’ space. For this reason, SRM techniques are typically showcased to image distinct biological structures, such as microtubules or nuclear pore complexes (NPC), that combine high labeling density with a high degree of spatial isolation, particularly along the *z*-axis. In contrast, densely stacked structures (e.g. chromatin) are often less well resolved due to contamination of the specific signal with blurred and scattered light, which raises the ‘noise floor’ in the Fourier domain (see Glossary) and thus reduces the effective resolution limit (cut-off frequency). Different SRM techniques deal better or worse with this challenge, but ultimately every technique will be defeated by a lack of contrast. Hence, experimentalists should make sure that a sample has a high ratio of specific labeling versus unspecific background fluorescence, that the system operates with low detector noise, and that the dynamic range is as high as possible while keeping photobleaching and phototoxicity in check.

## What are the benefits and/or downsides of linear versus non-linear microscopy methods?

In linear microscopy methods, the fluorescence emission intensity (*I*_emission_) is directly proportional to the excitation light intensity (*I*_excitation_). This means that as the intensity of the excitation light increases, the fluorescence intensity also increases in a linear fashion. This proportionality assumes that the system is operating within the linear range of the response of the fluorophore and that other factors, such as photobleaching and saturation, are minimal. The empirical assumption is that *I*_emission_∝*I*_excitation_. In contrast, in non-linear imaging modalities, the fluorescence follows a non-linear relationship. Although non-linear methods offer improved resolution and contrast, they require more complex equipment and higher excitation power.

**D.B.:** Answering this question requires a further subdivision of techniques. Techniques including STED, SMLM and non-linear SIM rely on non-linear photo-physical processes but can produce images that are linear (or approximately linear) in terms of the relationship between brightness and fluorophore concentration. These generally yield better resolution at the cost of increased illumination light dosage and potential for bleaching as well as higher instrumental cost and/or complexity but otherwise present no real downsides or caveats when compared with optically linear methods. Moreover, although commonly the case with non-linear methods, increased light dose is not a hard and fast rule. In cases of high background, the optical sectioning capability afforded by techniques such as STED can even mean that the dose required to achieve comparable SNR and resolution might be lower than that required by linear SIM.

SOFI (Dertinger et al., 2009) and related techniques, some machine-learning methods and poorly executed SMLM produce an image in which the relationship between brightness and the concentration of the underlying fluorophore is non-linear. These approaches are useful in some circumstances, but interpretation of the resulting data and any associated resolution claims require extreme care, as many of the assumptions that are typically made during image processing are invalid. SMLM can experience these pitfalls as signal localization density is increased, or because of poor experimental choices or sample characteristics. This can result in areas of the image that are incorrectly reconstructed due to localizations that were too close together or non-uniform background across the field of view. A similar problem occurs due to stochastic under-sampling, when either labeling density is too low or acquisition time is too short.

**C.E.:** The advantages of non-linear scanning approaches, such as multi-photon excitation, are deeper sample penetration with better confinement of the signal and photobleaching to the region of interest, yet they carry the disadvantage of lower signal levels and much higher photobleaching levels inside the immediate observation spot.

**R.F.:** There are different interpretations to this question. Non-linear techniques can include those that use a non-linear fluorophore response, such as STED and non-linear SIM, and techniques that, through post-processing, yield a non-linear response to the observed fluorescence intensity (e.g. SOFI). STED and non-linear SIM have a non-polynomial relationship to intensity, and as such their resolution is, in theory, unlimited. In practice, they face boundaries set by SNR and sample health. STED has proven itself to be a viable SRM technique, even for live-cell imaging. However, great care must be taken to ensure the cells can tolerate the high light intensities. I hope that we will see more non-linear SIM results in the future as it requires a surprisingly simple experimental setup. Exploiting the photophysical properties of fluorophores has great potential for live-cell imaging.

**R.H.:** Although valid and powerful approaches, non-linear methods often suffer from the need for high illumination intensities, which can sometimes cause super-linear bleaching. However, if these non-linear sample–response approaches are performed carefully, they are still linear in the sense that twice the sample concentration yields twice the brightness in the image. Apart from physical effects (self-quenching, steric fluorogenic effects, etc.) that might alter this and prevent reliable quantification, there are also microscopy methods, such as SOFI, that involve somewhat non-linear image processing (Heintzmann and Benedetti, 2006) such that the final image cannot be described by a single final PSF. Deconvolution, particularly using powerful assumptions on how valid samples should look (so-called ‘priors’), and deep learning-based methods also fall into this group. These methods are extremely useful for discovering new features and possibly for counting objects but should be used with great care when quantification of the emitted light is paramount.

**S.M.:** Non-linear techniques offer several advantages, such as enhanced resolution, reduced out-of-focus background and the ability to probe deeper into samples. These improvements arise from harnessing non-linear responses, making them particularly useful for imaging applications that require molecular-scale resolution or imaging targets away from the coverslip (e.g. dendritic spines or synapses in brain tissue). Ultimately, the choice between linear and non-linear methods depends on the scientific question being addressed and the specific imaging requirements.

**C.S.:** Linear methods are simpler, and effective for quantification, but are limited by resolution, imaging depth and background noise, whereas non-linear microscopy methods have the potential to achieve better resolution and penetration. However, it is important to note that non-linear techniques can be less predictable and robust due to the inherent challenges associated with higher illumination intensities, photobleaching and sample damage. The trade-off between improved imaging capabilities and these challenges means that non-linear microscopy methods can require careful optimization and specific experimental conditions to obtain reliable results. Researchers should be prepared to manage these factors and expect a longer lead time to harness the full potential of non-linear microscopy for achieving super-resolution and deeper imaging in their specific applications. The choice depends on the specific imaging goals, such as resolution, depth or quantification.

**H.S.:** Usually, when biologists say ‘quantitative’, they mean ‘linear’. Therefore, if estimating numbers or concentrations is important, it is useful to think about the set of conditions for which a microscopy technique produces an output signal that is linearly related to the concentration of labeled fluorophore. If you are more interested in determining the shape of a feature (e.g. for segmentation), linearity is likely a less important consideration.

**L.S.:** Non-linear SRM methods, including those incorporating deconvolution, offer the potential for increased, sometimes theoretically unlimited (although practically contrast-limited) spatial resolution. However, they can also introduce artifacts, such as the deletion of dim features and overrepresentation of bright features. One drawback of certain non-linear methods is that the resulting images no longer reflect the abundance or density of fluorophores in a linear manner. For instance, in SMLM methods that rely on extensive filtering of inherently non-linear blinking or photoswitching events, regions with high concentrations of fluorophores might have a larger fraction of discarded events, whereas events in less densely labeled areas or the background could be overrepresented, leading to unwanted bias shifts. In contrast, linear SIM exhibits a largely linear relationship between the number of fluorophores or target molecules and the intensity, making it suitable for intensity-based quantification.

## How does the potential of Ångstrom-resolution light microscopy compare to that of EM?

The increasing convergence of modulation-enhanced SMLM methods (Reymond et al., 2020), such as MINFLUX or advanced DNA-PAINT approaches, such as RESI (Reinhardt et al. 2023), which are capable of localization precisions below 1 nm, and cryogenic electron microscopy (cryo-EM) has ignited important discussions within the structural biology field (Balzarotti et al., 2017; Reinhardt et al., 2023; Weisenburger et al., 2017; Shaib et al., 2024). Although both methods are now often mentioned in the same breath, they represent distinct, non-overlapping techniques with unique application areas. Cryo-EM provides atomic details (structural resolution, see Glossary) of protein structures, whereas SRM excels for applications such as locating specific molecule species and/or target sites and their relative spatial relationships, tracking dynamics and colocalization of proteins *in situ*. This juxtaposition raises crucial questions. What should the definition of ‘Ångstrom-resolution’ be in SRM compared to in cryo-EM? How can these methods, which resolve such different biological features, be fairly compared? Furthermore, lack of standardized nomenclature in SRM for reporting performance exacerbates the challenge, especially for methods that claim to resolve protein structures at scales similar to those in cryo-EM (Shaib et al., 2024). Defining these standards and clarifying the goals of SRM in the context of cryo-EM will be essential for guiding the field toward more objective comparisons and future developments.

**C.S. and K.P.:** When compared to EM, particularly cryo-EM, an advantage of fluorescence-based SRM techniques approaching the Ångstrom scale is labeling specificity. However, disadvantages include sub-optimal label efficiency and distance between the dye and the biological site of interest. The reduced labeling efficiency can be mitigated by averaging multiple images (Heydarian et al., 2021), whereas addressing linker length needs a biochemical solution (Kompa et al., 2023; Erdmann et al., 2019). Some SRM methods (Table 1) can only visualize static structures, but MINFLUX is particularly useful in tracking applications, for example in tracking of kinesins and microtubules (Deguchi et al., 2023), because it reduces photobleaching of the fluorescent protein that is being tracked by efficiently using the available harvestable photons (photo budget). Here, it is not about creating an image, but about the information that is acquired (Kalisvaart et al., 2022; Smith et al., 2024). Going forward, correlative SRM and cryo-EM, capable of imaging the same biological structure under identical conditions, offer a pathway to calibrate and directly compare the effective resolution of each technique, helping to bridge the interpretational gap between them.

**C.E.:** Both techniques have produced outstanding results, but their power to resolve dense cellular structures remains to be shown. Further developments are also needed to determine what kind of information, complementary to cryo-EM, Ångstrom-resolution microscopy will bring. As a big supporter of correlative techniques, I have hope that complementary Ångstrom-resolution methods and cryo-EM data, along with increased experimental efforts (e.g. improved labeling methods), will generate improved information on molecular structures.

**H.S.:** Assessing Ångstrom-resolution claims should be done in a similar way to assessing more conventional techniques using known standards. For Ångstrom-resolution microscopy, what is the smallest distance (spatial frequency) that can be reliably resolved above the noise floor? Additionally, challenges of these methods include the density of labels, attachment chemistry, specificity of labeling, etc. However, the key advantage of Ångstrom-resolution microscopy over EM is its ability to offer molecular specificity.

**L.S.:** It is important to note that the claims of (sub-)nanometer or Ångstrom-resolution for hybrid SMLM methods typically refer to the localization precision of the fluorescent label and therefore cannot be directly compared with the structural resolution obtained by EM techniques. Furthermore, the practical application of Ångstrom-resolution techniques to resolving biological structures *in situ* is currently still limited. It will be interesting to see how the competition between the development of correlative super-resolution approaches, such as correlative light and (cryo)electron microscopy (cryoCLEM), and purely optical methods (with or without physical expansion of samples) will progress over the next few years. A key factor will be the concurrent advancement of labeling methods that can densely cover biological molecules to take advantage of the spatial resolution already achievable, for example, with MINFLUX and related techniques.

## How can one accurately assess image quality and deal with artifacts in SRM?

Image artifacts are a common theme in optical microscopy and are often exacerbated in SRM. These can be defined as deviations from ‘ideal’ images, closest to the ‘ground truth’, as obtainable under optimal conditions with a given technique. Root causes are spherical aberrations due to refractive index mismatches (e.g. between the immersion medium, the sample and its surrounding embedding medium, as well as from inhomogeneities within the sample), chromatic shifts, light scattering, out-of-focus blur, etc., to which different SRM methods might have different susceptibilities. In addition, method-specific artifacts exist particularly for those relying on computational reconstructions. In SIM, suboptimal sample preparation and/or reconstruction parameters might lead to ringing (halo effects around objects), overshooting (exaggerated brightness or darkness at edges), honeycomb pattern (grid-like distortions from 2D SIM illumination) and reconstructed noise (‘hammer stroke’) artifacts (Demmerle et al., 2017). SMLM methods heavily rely on filtering, which can easily lead to biased representations of sample features (Prakash, 2021, 2022).

**C.S. and K.P.:** The quality of an image is determined by multiple factors, including the microscope setup, sample preparation and image reconstruction techniques. Computational post-processing, such as deconvolution and denoising, play a significant role in improving image quality but can also introduce artifacts. These artifacts are often easier to detect with a solid understanding of the underlying algorithms. Artifacts related to sample preparation, data acquisition and reconstruction can be identified and corrected for (Ball et al., 2015; Smith et al., 2021; Li and Betzig, 2016). For SMLM, several software tools offer simulation-based analyses to aid in detecting and mitigating reconstruction artifacts (Costello and Cox, 2021). For neural network-based SRM methods, checkerboard pattern artifacts have been observed (Speiser et al., 2021). The primary challenge for SRM is discerning whether a new observation is a novel finding or an artifact. For this reason, a significant focus has been on correlative SRM, where applying two different super-resolution methods with similar resolution ranges on the same section of a sample under almost identical imaging conditions (Prakash, 2024).

**C.E.:** The moment one uses extensive data analysis for generating SRM data, one can expect artifacts. One way to identify artifacts is to apply different SRM techniques and check data between approaches. Another strategy is to compare different labeling or sample preparation approaches.

**R.H. and H.S.:** Artifacts typically arise from a disagreement between assumptions about the imaging conditions and reality. Systems that do not rely on image processing are typically less prone to artifacts than systems that need extensive image processing, where a violation of underlying assumptions can sometimes generate severe artifacts. SIM, in which hexagonal-shaped artifacts are often seen, is an example of the latter (Karras et al., 2019). Artifact types are typically closely linked to the system being used, and recognizing them is also a very system-specific task. It is good practice to confirm an observation made in one type of system using a different type of super-resolution system.

**L.S.:** Artifacts come in many shapes and forms and no microscopic image is truly free of them. When taken to the extreme, diffraction and its impact on resolution could be considered an artifact compared to the ground truth. Many artifacts, such as spherical, chromatic and sample-induced aberrations, nonspecific labeling, artifacts from low SNR or bleaching, and in the case of live-cell imaging, motion blur and cytotoxicity, affect all imaging methods to some extent. Other artifacts are specific to SRM methods that rely on post-processing, such as SMLM, SIM or any approach that includes non-linear deconvolution. In these cases, parameter values often need to be carefully selected to balance one type of artifact against another and find the best compromise. Understanding how a certain artifact affects data quality for quantitative evaluations and biological interpretation is crucial and requires appropriate controls. It is therefore extremely important for every experimentalist to know common sources of artifacts, how to identify them and how to apply appropriate countermeasures, whether in sample preparation, experimental settings or post-processing. Fortunately, there are many tools, resources and literature available to provide guidelines and support (Ball et al., 2015; Demmerle et al., 2017; Culley et al., 2018; Marsh et al., 2021).

## Conclusion

SRM has evolved from a technology-driven field into an indispensable tool for biological discovery. As outlined in this Perspective, different SRM modalities provide complementary advantages and cater to diverse applications in cellular and molecular biology. For instance, 3D SMLM has significantly contributed to elucidating cell surface structures, such as adhesion complexes and vesicle formation (Kanchanawong et al., 2010; Bertocchi et al., 2017). STED microscopy has been used very successfully in neurocytological applications, such as in imaging of dendrite spine and synapse organization (Ritter et al., 2015; Hruska et al., 2018), and in combination with fluorescence correlation spectroscopy (FCS) has revealed dynamic lipid organization in membranes (Eggeling et al., 2009; Honigmann et al., 2014). SIM and SMLM have become invaluable in revealing insights into various aspects of nuclear and chromosomal biology (Schermelleh et al., 2008; Kirmes et al., 2015; Prakash et al., 2015; Ricci et al., 2015; Boettiger et al., 2016; Bintu et al., 2018; Miron et al., 2020; Szabo et al., 2020; Ochs et al., 2019, 2024; Rodermund et al., 2021; Markaki et al., 2021) and have been proven powerful tools for studying organelle and cytoskeleton dynamics (Li et al., 2015; Guo et al., 2018). Various SRM approaches have provided crucial insights into centrosomes, NPCs, cytoskeletal filaments and other macromolecular assemblies (Xu et al., 2013; Stoldt et al., 2018; Stephan et al., 2020; Kleele et al., 2021; Szymborska et al., 2013; Yang et al., 2018). Finally, pixel reassignment methods offer easy extended-resolution imaging ‘at the click of a button’ and are an important bridge towards less routine higher resolving approaches.

A fundamental principle in microscopy is that there are no solutions; there are trade-offs. Each imaging technique has strengths and limitations and failing to recognize these trade-offs means failing to understand microscopy. Whether it is a compromise between resolution and speed, SNR and photobleaching, or precision and throughput, every experimental design must account for these constraints. Looking ahead, the integration of SRM with other advanced imaging methods, such as cryo-EM and ExM, will further expand its application range and offer even more biological insight. The same applies to more specialized methods, such as MINFLUX, as they become increasingly available for exploration by biologists. Furthermore, developments in computational approaches, including AI-based denoising and image restoration, promise to overcome existing limitations in resolution and contrast. Ultimately, as SRM becomes increasingly accessible and automated, its role in deciphering fundamental biological mechanisms will only grow, marking a definitive shift from a technical novelty to a transformative force in life sciences research.

As more studies demonstrate the value of SRM for biological research, it has become clear that different SRM methods cover different application ranges with distinct application ‘sweet spots’ and that there is no one-size-fits-all solution. However, it is evident that the time when biological SRM images of well-known structures were used primarily to showcase a method or to add a ‘nice image’ is coming to an end and the era in which SRM is central to discovering new biology is firmly underway.
